# Comparative Study of Transradial Versus Transfemoral Route Percutaneous Coronary Intervention in Acute ST-Elevation Myocardial Infarction

**DOI:** 10.7759/cureus.32983

**Published:** 2022-12-27

**Authors:** Prashant Kashyap, Chigulapalli Sridevi, Susheel Kumar Malani, Vivek V Manade

**Affiliations:** 1 Department of Cardiology, Dr DY Patil Medical College, Pune, IND

**Keywords:** transfemoral access, transradial approach, st-elevation myocardial infarction (stemi), percutaneous coronary intervention, cardiovascular diseases (cvd)

## Abstract

Introduction

Percutaneous coronary intervention (PCI) is the first choice of treatment for myocardial infarction (MI). However, entry site failure is still one of the major complications faced by the interventionist. Hence the present study compared the efficacy and complications of radial and femoral approaches in PCI in ST-elevation myocardial infarction (STEMI).

Methods

A hospital-based prospective study was conducted on patients with acute STEMI. A total of 100 patients were enrolled that were randomly divided into two groups of 50 each, i.e., patients that had undergone PCI by radial approach (N=50) and those who had undergone PCI by femoral approach (N=50).

Results

The male-to-female ratio was 1.5:1 and 1.6:1 in the transradial (TR) and transfemoral (TF) groups, respectively. With respect to age, both the groups were dominated by the age group of 50-60 years, with 42% in the TR group and 34% in the TF group having an age >60 years. The mean access time, fluoroscopy time, and procedural time in the TR group were 6.0 ± 0.7 minutes, 5.9 ± 0.6 minutes, and 29.55 ± 0.9 minutes, respectively. In the TF group, the mean access time, fluoroscopy time, and procedural time were 5.1 ± 0.5 minutes, 5.5 ± 0.7 minutes, and 26.7 ± 2.1 minutes, respectively. In the TR group, ecchymosis and loss of radial pulse were observed in 10% of the patients, thrombophlebitis in 4%, and bleeding complications in 2%. While in the TF group, ecchymosis was observed in 26%, followed by thrombophlebitis (24%), minor hematoma, and bleeding complications (14%).

Conclusion

The present study emphasizes the use of radial access in patients with ST-segment elevation acute coronary syndrome, as this approach was associated with significant clinical benefits. Moreover, bleeding complications were more in patients undergoing TF intervention.

## Introduction

Cardiovascular diseases (CVD) have superseded cancer and have become the leading cause of death in India since the dawn of the century [1]. Indians, especially those in middle age, are more susceptible to CVD than Europeans [2,3]. For instance, 23% of CVD deaths occur before the age of 70 in the western population, whereas it is as high as 52% in the Indian population [4]. The mortality rate attributed to CVD in India is high compared to the middle- and high-income per capita countries [5,6]. The WHO predicts that India will lose $237 billion in lost productivity spending on health expenditures due to the encumbered CVD situation [7]. There are various treatment options for myocardial infarction (MI), and percutaneous coronary intervention (PCI) is the first choice of treatment among cardiologists. PCI is performed by using a catheter that is accessed into the arterial system through the femoral, brachial, or radial artery [8]. One of the complications during the procedure is the inability of a coronary guidewire to cross an occlusion. It has been three decades since the transfemoral (TF) approach dominated PCI development, but radial access has recently become more popular due to perceived safety benefits [9]. However, the data regarding the safety and efficacy of transradial (TR)-PCI compared with TF-PCI in ST-elevation myocardial infarction (STEMI) diseases are limited, and that too very scarce data is available among Indian patients. Hence the present study was designed to compare the efficacy and complications of radial and femoral approaches in PCI in STEMI.

## Materials and methods

The present study was a prospective study conducted in the Department of Cardiology of a tertiary care hospital, Dr. DY Patil Hospital, Pune. A total of 100 consecutive patients with acute STEMI that presented to the ED and underwent PCI were chosen for the study. Of these 100 patients, 50 underwent radial, and 50 underwent femoral approaches.

Inclusion criteria

Inclusion criteria included patients with acute STEMI who underwent PCI.

Exclusion criteria

Exclusion criteria included patients with (1) Renal insufficiency (serum creatinine >2.0mg/dl); (2) Patients with severe sepsis, local site infection, prior contrast allergy, or severe intrinsic/iatrogenic coagulopathy (INR > 2); (3) An abnormal modified Allen's test (for TR route); (4) Peripheral vascular disease (Iliofemoral disease) (for TF route); and (5) Patients with prolonged pulseless cardiogenic shock.

Method of study

The current study included all patients with acute STEMI who met the inclusion and exclusion criteria. Data on the patient's comprehensive histories and medical examinations were obtained. Height and weight measurements were taken in order to calculate BMI. Blood pressure was measured in the right upper limb in the sitting posture with an adequate size cuff. An ECG test was done at the time of presentation, and a serial ECG later. Hemoglobin (Hb), total leucocyte count (TLC), differential leukocyte count (DLC), peripheral blood film (PBF), fasting/random blood sugar (FBS/RBS), lipid profile, serum CK-MB, blood urea, and serum creatinine were also recorded.

Procedural anticoagulation was established in the patients by administering an unfractionated heparin bolus at a dosage of 70 UI/kg, supplemented during the surgery to maintain an active clotting time of 250 s. According to the institution's management policy, the choice of additional periprocedural antithrombotic medications (e.g., glycoprotein IIb/IIIa inhibitors or bivalirudin) or various revascularization techniques (e.g., thrombectomy, direct stenting) was left to the operators. All anticoagulants were stopped at the end of the surgery unless clinically contraindicated, and glycoprotein IIb/IIIa inhibitor boluses were followed by a > 12-h infusion. All patients were pre-treated with acetylsalicylic acid plus a loading dose of clopidogrel (300-600 mg) and were discharged on dual antiplatelet and HMG CoA reductase inhibitors (atorvastatin 80 mg) medication for more than 12 months, depending on the stent placed.

A physician checked bilateral femoral and radial pulses prior to the surgery. In particular, Allen's test was conducted twice on both hands to rule out inadequate ulnar collateral circulation. If an abnormal Allen's test result was obtained, additional investigation with pulse oximetry or plethysmography is not prohibited but is not suggested either to avoid constant time delay. Radial pulse and Allen's test were measured in patients with cardiogenic shock after intra-aortic balloon placement or particular pharmacological therapy (i.e., inotropic medication delivery). In order to deem a patient eligible for the TR technique, Allen's test demonstrating a well-functioning ulnar artery was required. Following local anesthesia with 2% lidocaine, the radial artery was cannulated with a 19-gauge needle, through which a 0.022′′ guidewire was advanced, and a 6F radial sheath (Terumo, Japan) was inserted. A vasodilating medicinal cocktail of 5 mg dilzem and 100 micrograms of nitroglycerine were administered. External compression, either with the TR band (Terumo, Japan) or Radistop, was used to produce hemostasis. Following local anaesthetic with 2% lidocaine, a 6F sheath was advanced over a 0.035′′ guidewire using the Seldinger method for the TF approach. The usage of closing devices was at the discretion of the operator. Unless their clinical status indicated otherwise, participants in the TR group were permitted to ambulate 1 hour after intervention and 12-24 hours in the femoral group.

The patient data were collected in a case record sheet and later compiled using Microsoft Excel. All the results were analyzed by SPSS software version 17.0. The Chi-square test and Mann-Whitney U test were used for the assessment of the level of significance. A p-value of less than 0.05 was considered statistically significant. Informed consent was obtained from the enrolled patients. The study was also approved by the Institutional Research and Recognition Committee of Dr. DY Patil Vidyapeeth, Pune (DPU/R&R(M)/437/2021; Dated May 25, 2021).

## Results

Out of 100 participants, 61% were males and 39% were female. The male-to-female ratio was 1.5:1 and 1.6:1 in the TR and TF groups, respectively. With respect to age, both the groups were dominated by the age group of 50-60 years, with 42% in the TR group and 34% in the TF group age with age >60 years. A total of 42% in the TR group and 34% in the TF group had age >60 years. In terms of comorbidities, in the TR group, 32% of the patients had dyslipidemia, 26% of the patients had diabetes, 38% of patients had hypertension, and 20% of the patients had a family history of coronary artery disease (CAD). While in the TF group, 28% of the patients had dyslipidemia, 36% had diabetes, 40% had hypertension, and 18% had a family history of CAD. In the TR group, 40% had double vessel disease while 24% had triple vessel disease, whereas it was 42% and 24%, respectively, in the TF group (Table 1). The mean access time, fluoroscopy time, and procedural time in the TR group were 6.0 ± 0.7 minutes, 5.9 ± 0.6 minutes, and 29.55 ± 0.9 minutes, respectively. In the TF group, the mean access time, fluoroscopy time, and procedural time were 5.1 ± 0.5 minutes, 5.5 ± 0.7 minutes, and 26.7 ± 2.1 minutes, respectively. The mean length of hospital stays in the TR and TF groups were 2.9 ± 0.6 days and 4.2 ± 0.7 days, respectively (Table 2). There was no significant difference between any of the investigated lab parameters like hemoglobin, Troponin T, creatinine, total cholesterol, HbA1c, etc. (Table 3). In the TR group, ecchymosis and loss of radial pulse were observed in 10% of the patients, thrombophlebitis in 4%, and bleeding complications in 2%. While in the TF group, ecchymosis was observed in 26%, followed by thrombophlebitis (24%), minor hematoma, and bleeding complications in 14%. Also, major hematoma and pseudoaneurysm were observed in 8% of the patients in the TF group (Table 4). A total of 90% of the patients in the TR group and 92% of the patients in the TF group were satisfied with the procedure with no significant association between the two groups (P>0.05; Table 5).

**Table 1:** Baseline characteristics and demographics of the subjects in the respective groups. CVD: Cardiovascular diseases; CAD: Coronary artery disease.

Variable	Transradial group (N=50)	Transfemoral group (N=50)	P-value
Gender			
Male	30 (60%)	31 (62%)	0.838
Female	20 (40%)	19 (38%)	
Age group			
<50	5 (10%)	4 (8%)	0.605
50-60	24 (48%)	29 (58%)	
>60	21 (42%)	17 (34%)	
CVD risk factors			
Smoking	15 (30%)	17 (34%)	0.668
Dyslipidemia	16 (32%)	14 (28%)	0.663
Diabetes	13 (26%)	18 (36%)	0.280
Hypertension	19 (38%)	20 (40%)	0.838
Family history of CAD	10 (20%)	9 (18%)	0.799
Vessel involvement			
0-Vessels	2 (4%)	3 (6%)	0.949
1-Vessels	16 (32%)	14 (28%)	
2-Vessels	20 (40%)	21 (42%)	
3-Vessels	12 (24%)	12 (24%)	

**Table 2:** Descriptive statistics of the subjects in respective groups.

Variable	Transradial group (N=50)	Transfemoral group (N=50)	P-value
Access time (min)	6.006 ± 0.724	5.14 ± 0.459	0.0001
Fluoroscopy time (min)	5.896 ± 0.624	5.45 ± 0.734	0.0015
Procedural time (min)	29.55 ± 0.9	26.76 ± 2.1	0.0001
Hospital stay (Days)	2.92 ± 0.695	4.14 ± 0.782	0.0001

**Table 3:** Lab parameters of the subjects in respective groups. CK-MB: Creatine kinase-MB; HDL: High-density lipoprotein; LDL: Low-density lipoprotein.

Lab parameters	Transradial group (N=50)	Transfemoral group (N=50)	P-value
Hemoglobin (mg/dl)	14.8 ± 1.6	14.5 ± 1.8	0.38
CK-MB (mg/dl)	45 ± 60	29 ± 54	0.164
Troponin T (ng/dl)	1.4 ± 3.2	1.3 ± 2.4	0.86
Creatinine (mg/dl)	0.9 ± 0.7	0.8 ± 0.5	0.413
Total cholesterol (mg/dl)	179 ± 45	185 ± 30	0.434
Triglycerides (mg/dl)	120 ± 85	113 ± 75	0.663
HDL	40 ± 12	38 ± 10	0.367
LDL	115 ± 35	113 ± 19	0.723
Serum Glucose	160 ± 63	171 ± 68	0.4
HbA1c	6.5 ± 1.5	6.6 ± 1.8	0.763

**Table 4:** Complications observed in the subjects of the respective groups.

Complications	Transradial group (N=50)	Transfemoral group (N=50)	P-value
Major hematoma	0	4 (8%)	0.041
Minor hematoma	0	7 (14%)	0.006
Bleeding complications	1 (2%)	7 (14%)	0.027
Thrombophlebitis	2 (4%)	12 (24%)	0.004
Ecchymosis	5 (10%)	13 (26%)	0.037
Pseudo aneurysm	0	4 (8%)	0.041
Loss of radial pulse	5 (10%)	0	0.022
Loss of femoral pulse	0	0	-

**Table 5:** Post-procedural satisfaction of the patients in the respective groups.

Satisfaction to procedure	Transradial group (N=50)	Transfemoral group (N=50)	P-value
Yes	45 (90%)	46 (92%)	0.924
No	5 (10%)	4 (8%)	
Total	50 (100%)	50 (100%)	

## Discussion

In the present study, male predominance was observed in both groups. The male:female ratio in both the TR and TF groups was almost similar, and it was found to be 1.5:1. Bhat FA et al. also reported that male:female ratio in the TR approach as 2.8:1 and while in the TF approach, it was 2.0:1 [10]. Similar findings were found in studies done by Ferdinand K et al. and Li H et al. [11,12]. The majority of the population in the TR and TF groups were in the fifth decade of life. It was found to be 48% and 58%, respectively. There was no significant difference between the two groups in terms of gender and age distribution (P>0.05). The mean age of the patients in the TR group was 60.7 ± 10.5 years, while the mean age of the subjects in the TF group was 62.7 ± 7.8 years. There was no significant statistical difference between the two groups (P>0.05; Table 1). In a study by Bhat FA et al., the mean age of the participants in the TR group was 61.8 ± 6.6 years, and 60.6 ± 10 years in the TF group [10]. Similar findings were observed in a study done by Li H et al. where the mean age was 62.7 ± 12.2 years and 61.8 ± 12.4 years in the TR and TF groups [12] [13-14].

In the present study, the mean hospital stay was 2.9 ± 0.7 days and 4.1 ± 0.8 days in the TR and TF groups, respectively, and the difference was statistically significant (P=0.001). Irrespective of the complications, patients in the TF group had a long hospital stay than the TR group (Table 2). These findings were in concordance with the study by Bhat FA et al., who reported the mean length of hospital stay to be 3.6 ± 1.3 days and 4 ± 1.1 days for the TR and TF groups, respectively [10]. Similar findings were observed in a study done by Kiemeneij F and Laarman GJ, Vefali V and Arslan U, and Tewari S et al. [11,15-16].

Hemorrhagic complications form crucial and problematic risk factors for an unfavorable outcome. Because of this, more focus was kept to reduce all avoidable iatrogenic hemorrhagic complications. The use of a TR approach for acute patients undergoing early invasive treatment played a significant role in the prevention of access site-related bleeding as compared to the TF approach. In our study, in the TR group, the bleeding complication was observed only in 2% of the population, thrombophlebitis was observed in 4% of the subjects, ecchymosis in 10% of the subjects, and loss of radial pulse in 10%. Whereas in the TF group, major hematoma was observed in 8% of the subjects, minor hematoma in 14%, bleeding complication in 14%, thrombophlebitis in 24%, ecchymosis in 26%, and pseudoaneurysm in 8% (Table 4). The difference was statistically significant between the two groups regarding the occurrence of complications (P<0.05). The complications regarding procedures were more in the TF group than in the TR group. Similar observations were putforth by Bhat FA et al., and observed statistical significance between the two groups with more complications in the TF group [10]. In a study done by Choussat R et al., bleeding at the access site was noted in 7.4% of the TF group, whereas no patients had access site bleeding among the TR group [17]. A study by Agostoni P et al. had a significantly low complication rate but a higher rate of procedure failure in the TR group [18]. The findings in our study were consistent with the findings of Li H et al., Tewari S et al., Adnan SM et al., and Marti V et al. [12,16,19-20].

## Conclusions

Primary angioplasty is usually done either through the TF or TR approaches. This study signifies and highlights the benefits of using radial access in patients presenting with an acute coronary syndrome, STEMI. It also shows fewer incidences of bleeding complications compared to the femoral route. Thus, if possible, TR approach should become the recommended approach in patients with STEMI.
